# Prevalence of chronic spinal pain and identification of associated factors in a sample of the population of São Paulo, Brazil: cross-sectional study: Prevalência de dores de coluna crônicas e identificação de fatores associados em uma amostra da população da cidade de São Paulo, Brasil: estudo transversal

**DOI:** 10.1590/1516-3180.2016.0091310516

**Published:** 2016-09-26

**Authors:** Jidiene Dylese Presecatan Depintor, Eduardo Sawaya Botelho Bracher, Dayane Maia Costa Cabral, José Eluf-Neto

**Affiliations:** I DC, MSc. Associate Professor, Instituto Paulista de Pós-Graduação (IPPG), São Paulo, SP, Brazil.; II MD, DC, PhD. Private Practice, Axis Clinica de Coluna, São Paulo, SP, Brazil.; III DC, MSc. Associate Professor, Universidade Anhembi Morumbi, São Paulo, SP, Brazil.; IV MD, PhD. Professor, Department of Preventive Medicine, Faculdade de Medicina da Universidade de São Paulo (FMUSP), São Paulo, SP, Brazil.

**Keywords:** Back pain, Chronic pain, Low back pain, Prevalence, Cross-sectional studies, Dor nas costas, Dor crônica, Lombalgia, Prevalência, Estudos transversais

## Abstract

**CONTEXT AND OBJECTIVE:**

Chronic spinal pain, especially low-back pain and neck pain, is a leading cause of years of life with disability. The aim of the present study was to estimate the prevalence of chronic spinal pain among individuals aged 15 years or older and to identify the factors associated with it.

**DESIGN AND SETTING:**

Cross-sectional epidemiological study on a sample of the population of the city of São Paulo.

**METHOD:**

Participants were selected using random probabilistic sampling and data were collected via face-to-face interviews. The Hospital Anxiety and Depression Scale (HADS), EuroQol-5D, Alcohol Use Disorders Identification Test (AUDIT), Fagerström test for nicotine dependence and Brazilian economic classification criteria were used.

**RESULTS:**

A total of 826 participants were interviewed. The estimated prevalence of chronic spinal pain was 22% (95% confidence interval, CI: 19.3-25.0%). The factors independently associated with chronic spinal pain were: female sex, age 30 years or older, schooling level of four years or less, symptoms compatible with anxiety and high physical exertion during the main occupation. Quality of life and self-rated health scores were significantly worse among individuals with chronic spinal pain.

**CONCLUSION:**

The prevalence of chronic spinal pain in this segment of the population of São Paulo was 22.0%. The factors independently associated with chronic pain were: female sex, age 30 years or older, low education, symptoms compatible with anxiety and physical exertion during the main occupation.

## RESUMO

**CONTEXTO E OBJETIVO:**

A dor de coluna crônica, especialmente dor lombar e cervical, é uma causa importante de anos de vida com incapacidade. O objetivo deste estudo foi estimar a prevalência de algias vertebrais crônicas em indivíduos com 15 ou mais anos de idade e identificar fatores associados.

**TIPO DE ESTUDO E LOCAL:**

Estudo epidemiológico de corte transversal em uma amostra da população da cidade de São Paulo

**MÉTODO:**

A seleção de participantes foi feita por amostragem probabilística aleatória e a colheita de dados, por entrevistas presenciais. Foram utilizadas a escala hospitalar de ansiedade e depressão (HADS), o EuroQol-5D, o teste de identificação de desordens devido ao uso de álcool (AUDIT), o teste de Fagerström para dependência de nicotina e o critério de classificação econômica Brasil.

**RESULTADOS:**

Um total de 826 participantes foi entrevistado. A prevalência de algias vertebrais crônicas foi estimada em 22% (intervalo de confiança, IC 95%: 19,3-25,0). Os fatores independentemente associados com algias vertebrais crônicas foram: sexo feminino, 30 ou mais anos de idade, quatro anos ou menos de escolaridade, sintomas compatíveis com ansiedade e esforço intenso físico durante a ocupação principal. Participantes com algias vertebrais crônicas apresentaram escores de qualidade de vida e autoavaliação de saúde significativamente piores.

**CONCLUSÃO:**

A prevalência de algias vertebrais crônicas em um segmento da população de São Paulo foi de 22%. Os fatores independentemente associados à dor crônica foram: sexo feminino, idade igual ou superior a 30 anos, baixa escolaridade, sintomas compatíveis com ansiedade e esforço físico durante a ocupação principal.

## INTRODUCTION

Spinal pain is one of the most commonly reported musculoskeletal conditions.^1^ It has been estimated that 5-10% of cases of spinal pain become chronic^2^^,^^3^ and one fifth lead to pain-related disability one year after the first pain episode.^4^ Low-back pain and neck pain are the biggest and fourth biggest causes of years of life with disability worldwide, respectively, and the prevalence of neck pain is surpassed only by major depressive disorder and other musculoskeletal disorders.^5^

The International Association for the Study of Pain (IASP) defines chronic pain as pain that persists past the normal time of tissue healing. For nonmalignant pain, three months has been suggested as the most convenient point of division between acute and chronic pain. Chronic pain is a complex syndrome that involves biological, cognitive and lifestyle components.^6^^,^^7^ The American College of Rheumatology classification criteria for fibromyalgia define chronic widespread pain (CWP) as pain in the left and right sides of the body, above and below the waistline, together with axial skeletal pain.^8^

Reviews of the literature on chronic pain have indicated that the prevalences of chronic neck pain, upper back pain and low-back pain range from 14.5% to 51%,^9^^,^^10^^,^^11^^,^^12^^,^^13^^,^^14^^,^^15^ 10% to 20%^11^^,^^12^^,^^16^ and 15% to 45%,^1^ respectively. In Brazil, one study reported that the prevalence of chronic spinal pain (CSP) was 19%,^17^ and three other Brazilian studies reported prevalences of low-back pain ranging from 4.0 to 14.7%.^18^^,^^19^^,^^20^

Cultural and socioeconomic differences and distinct criteria for classifying chronic pain have been described as factors affecting the prevalence estimates for chronic pain.^21^^,^^22^ Several studies have used the duration of pain as the sole definition of chronic pain, and while most studies have defined chronic pain as pain that lasts for three months,^9^^,^^11^^,^^12^^,^^14^^,^^23^^,^^24^ others have considered it to be pain that persists for six or more months.^10^^,^^20^^,^^25^ Some studies have also included additional criteria for CSP, such as the presence of pain episodes over the last month and a score greater than or equal to 5 on a 0-10 visual analogue pain scale.^25^^,^^26^

Chronic spinal pain, especially low-back and neck pain, is usually associated with other painful conditions^27^^,^^28^ and psychological disorders.^3^^,^^29^ Female sex, greater age, low education levels, low socioeconomic status, anxiety and depression are commonly associated with CSP.^14^^,^^18^^,^^19^^,^^20^^,^^23^

Chronic spinal pain is a common symptom within the community and is associated with a significant impact on health. Understanding the epidemiology and impact of CSP is essential in developing public policies aimed towards prevention of spinal pain and health promotion.^30^

## OBJECTIVE

In this study, we estimated the prevalence of CSP among individuals aged 15 years or older and identified the factors associated with it. We also compared the health-related quality of life of individuals with and without CSP and estimated the prevalence of CWP among individuals with CSP.

## METHOD

This was a cross-sectional epidemiological study conducted in the central-western area of the city of São Paulo, Brazil, which is covered by the Family Health Program (FHP). The study was approved by the Research Ethics Committee of the University of São Paulo Medical School, the Research Ethics Committee of the São Paulo Municipal Health Department and the Research Ethics Committee of the Hospital Irmandade da Santa Casa de Misericórdia de São Paulo, which manages the "Dr. Alexandre Vranjac" Teaching Healthcare Center in Barra Funda, São Paulo. Permission to use the EuroQol-5D instrument was granted by the EuroQol group. All families residing in the study area were registered at this healthcare center.

### Population and sample

The community consisted of 8,052 individuals grouped into 2,549 families. Households were divided into 17 geographically defined micro-areas, each consisting of approximately 150 households. For this study, the prevalence of chronic spinal pain was estimated at 16%, based on a recent study conducted at a primary healthcare unit in São Paulo.^31^ The sample size was estimated as 482 individuals, considering a sampling error of 3% and a 95% confidence interval, but was then raised by 30% due to the expected losses, to a total of 627 individuals. Data collection for this study was conducted in conjunction with another study that evaluated the prevalence of chronic pain.^32^ Thus, 820 individuals were to be interviewed.

Participants were selected using a probability sampling method. In each micro-area, a number of households proportional to the size of the micro-area was selected using the random number generator function in the Excel software, version 2010.11. The Kish method was used to select one individual aged 15 years or older within each household. This method uses a pre-assigned table for each household, in which all residents are listed based on age and sex, relative to the head of the household. The member within the household to be interviewed is previously selected from the table to which the household has been assigned.^33^

Individuals of both sexes aged 15 years or older and registered with the FHP at the Barra Funda Healthcare Center were eligible to participate. Those who were unable to answer the questions during the interview, for whatever reasons, were not included in the study. Each household was visited up to four times, at different hours of the day and on different days of the week, in order to maximize the chance of contact with the selected participant. When the selected participant could not be reached after four visits, a person from another household was selected, using the same method described above. Re-draws were made to replace selected participants who were not interviewed.

A large number of foreigners from Bolivia, Paraguay and South Korea were found to be residing in the study area. Because of the language barrier and their reluctance to participate in the interview owing to their likely irregular status in the country, this group of individuals was excluded from the study.

### Data collection and instruments

Home interviews were conducted by two authors and by previously trained undergraduate students. A pilot study was conducted at a university practice ambulatory clinic to train interviewers and potentially improve the questionnaire.

**Figure 1:**
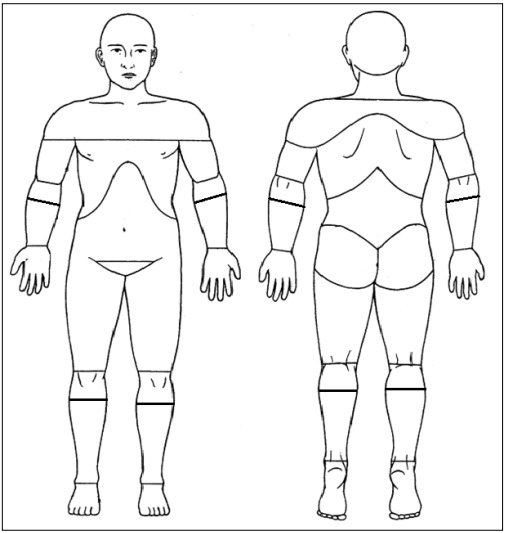
Pain diagram.

In accordance with the criteria from the International Association of Pain, chronic pain was defined as persistent pain for three or more months.^6^ In order to avoid selecting participants with low-frequency chronic pain, participants needed to report at least one pain episode in the previous month. Respondents with chronic pain were asked to indicate all their painful regions, by marking them on a diagram representing the front and back views of a human figure. We used a modified version of the Brief Pain Inventory, which originally divided the human body into 45 regions.^34^ In our study, the diagram was divided into 59 regions (Figure 1).

Respondents were asked to indicate the main location of pain, i.e. the area that hurt the most, by marking the diagram in Figure 1 with an arrow. Individuals with CSP were defined as those who indicated areas corresponding to the cervical, thoracic or lumbar regions, whether or not those were the main sites of pain.

Low-back pain was defined as pain localized in the region bounded by the twelfth rib, superiorly; the gluteal line, inferiorly; and the anterior axillary line, anteriorly. Neck pain was defined as pain localized in the region bounded by the lower edge of the occipital bone, superiorly; the spine of the scapula, inferiorly; and the anterior edge of the sternocleidomastoid muscle, anteriorly. Upper back pain was defined as pain in the posterior part of the chest between the first thoracic vertebrae and the upper contour of the trapezius muscle, superiorly; the twelfth thoracic vertebrae and the lower edge of the twelfth ribs, inferiorly; and the right and left axillary line, laterally.^35^

Chronic widespread pain (CWP) was defined as pain in the left and right side of the body, above and below the waistline. Axial skeletal pain was defined as pain in any of the following regions: neck, anterior or posterior part of the chest, or lower back^8^. Participants with chronic pain who did not have CWP were classified as having chronic regional pain (CRP).

The interview included questions asking for personal and sociodemographic information and administration of a pain questionnaire. Information on the presence of comorbidities was obtained through self-reporting. Additionally, four health assessment scales that had previously been validated and culturally adapted to the Brazilian cultural context, and one socioeconomic status scale, were applied.

Symptoms consistent with anxiety and depression were assessed using the Hospital Anxiety and Depression Scale (HADS).^36^ HADS was developed to assess non-psychiatric patients in different populations and has 14 items, seven of which relate to anxiety and seven to depression.^37^^,^^38^ Each item on the questionnaire was scored from 0 to 3 for a maximum score of 21 for either anxiety or depression. In our study, a cutoff of 9/21 points was established for symptoms of either anxiety or depression.^37^

Health-related quality of life was assessed by means of the EuroQol-5D (EQ-5D) instrument. EQ-5D includes questions about the following five dimensions: mobility, self-care, usual activities, pain/discomfort and anxiety/depression. The final score (EQ-index) combines the five dimensions and ranges from 0 (worst quality of life) to one (best quality of life). In addition, the respondent's self-rated health is recorded on a visual analogue scale (VAS) numbered from 0 to 100, where 100 means the 'best imaginable health state' and 0 means the 'worst imaginable health state'.^39^

The Fagerström Test for Nicotine Dependence (FTND) was used to assess the severity of nicotine dependence. This instrument contains six questions and respondents are assigned to one of five dependence levels.^40^^,^^41^

Alcohol use was assessed using the Alcohol Use Disorders Identification Test (AUDIT). AUDIT consists of 10 questions: three about hazardous alcohol use, three about dependence symptoms and four about harmful alcohol use. The final scores are grouped into four levels of risk.^42^

The participants' socioeconomic status was assessed by means of the Brazilian Economic Classification Criteria (CCEB), which classify the population into five socioeconomic categories from A to E, based on ownership of a range of durable assets and the head-of-household's education level.^43^

The participants answered all of these questionnaires, except the FTND, which was answered by smokers only.

### Statistical analysis

Descriptive analyses on the median, mean, standard deviation and percentage were used to establish the demographic and clinical characteristics of the sample. The CSP prevalence and its 95% confidence interval (CI) were determined. The association between CSP and the selected variables was estimated using prevalence ratios and their 95% CI.

We used a Cox regression model with constant time and robust variance.^44^ Cox regression is commonly used to analyze time-to-event data. When a constant risk period (time = 1) is assigned to all subjects, the hazard ratio estimated by Cox regression equals the prevalence ratio in cross-sectional studies.^45^

In bivariate analyses, statistical associations were determined by means of the log-rank test. For ordinal variables, we used the chi-square test for trend. Variables with a P-value < 0.20 in bivariate analyses were selected for multivariate analysis. Multivariate models were constructed by adding variables one at a time through forward stepwise addition, starting from the variable with the lowest P-value, followed by the other variables with P < 0.20. Variables with a P-value < 0.05 according to the maximum likelihood ratio test were retained in the final model. Lastly, we estimated the PR and 95% CI for each variable in the final model. Data were considered significant at P < 0.05.

The analyses were performed using the STATA 13.0 software (StataCorp LP, College Station, Texas, USA).

## RESULTS

### Characteristics of the source population and response rate

A total of 6,297 individuals aged 15 years or older were included, and most of them (3,666; 58.2%) were women. A total of 1,385 households (54.3% of the registered families) were selected to participate in the study, and one person from each household was selected for the interview. Of the selected individuals, 559 were not interviewed for the following reasons: they were ineligible (n = 277), were not located (n = 220), declined participation in the interview (n = 60) or were deemed dangerous to be interviewed (n = 2). The ineligible individuals were considered thus because they had moved (n = 192), were foreigners (n = 64), were incapable of answering (n = 15), or had died (n = 6). Thus, approximately three quarters of all the eligible individuals selected were interviewed (n = 826; 74.5%). Re-draws were made to replace all the eligible participants who had not been interviewed.

### Characteristics of the sample

In total, 826 individuals were interviewed between December 2011 and February 2012. The respondents' mean age was 51.4 ± 19.3 years. Most respondents were women (69.0%), single (62.2%) and had completed eight or more years of education (55.2%). Nearly half (48.9%) of the respondents reported suffering from at least one illness, 50.8% were employed at the time of the interview and most (93.7%) reported performing no hard physical activity during the workday. The vast majority of the respondents were of socioeconomic levels B or C (86%) (Table 1).

**Table 1:**
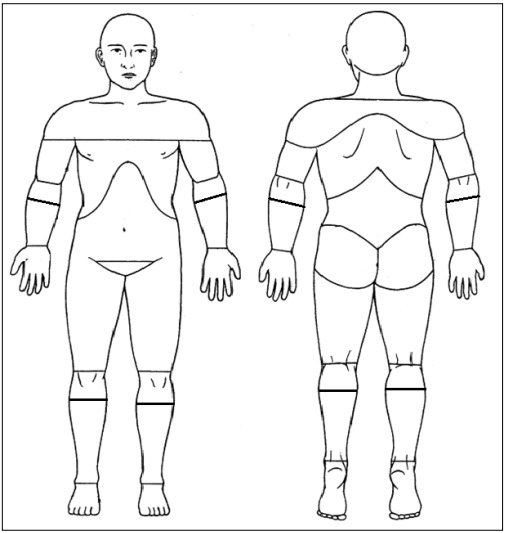
Characteristics of the sample

*5 individuals without data.

Symptoms consistent with anxiety and depression were observed in 189 (22.9%) and 96 (11.6%) respondents, respectively. Only 2.2% (14/637) of the individuals without anxiety symptoms had depression, whereas 43.4% (82/189) of the respondents with anxiety symptoms had depression. Most respondents were non-smokers (81.1%) and only 7.6% had high or very high nicotine dependence. Possible alcohol dependence, harmful use of alcohol or hazardous drinking was observed in 8.5% of the respondents (Table 1).

### Prevalence and characteristics of chronic back pain

**Figure 2:**
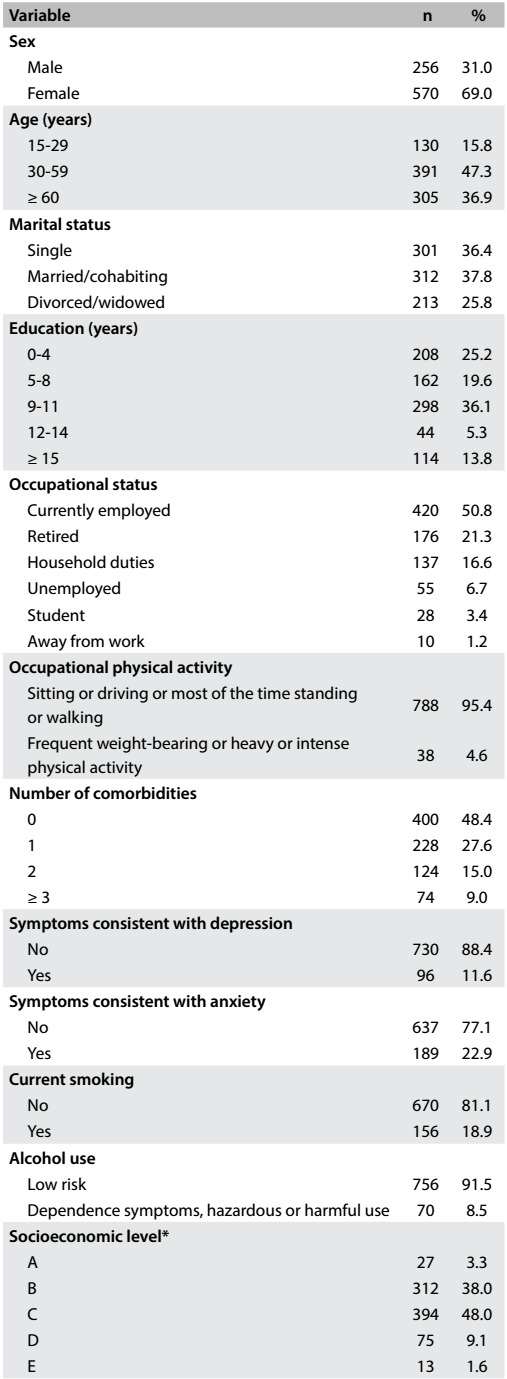
Prevalence of chronic spinal pain according to location.

Chronic spinal pain, defined as persistent pain in the cervical, thoracic or lumbar spine lasting three or more months and at least one pain episode in the last month, was reported by 182 individuals, corresponding to a prevalence of 22.0% (95% CI: 19.3-25.0%). The prevalence of CSP was significantly higher among women (25.8%; 95% CI: 22.2-29.6%) than among men (13.7%; 95% CI: 9.7-18.5; P < 0.001). Chronic low-back pain was reported by 152 individuals, corresponding to a prevalence of 18.4% (95% CI: 15.8-21.2%). Additionally, upper back pain and neck pain were reported by 56 and 47 individuals, respectively, corresponding to prevalences of 6.8% (95% CI: 5.2-8.7%) and 5.7% (95% CI: 4.2-7.5%), respectively. The sum of individuals with cervical, thoracic or lumbar pain exceeded the number of individuals with CSP because 54 respondents reported pain in more than one area (Figure 2). The individuals with CSP indicated 7.3 ± 7.2 painful regions and the mean duration of pain was 6.6 ± 8.6 years (median: 4.0 years.).

The prevalences of CRP and CWP among the individuals with CSP were 16.7% (95% CI: 14.2-19.3%) and 5.3% (95% CI: 3.8-6.9%), respectively. More than three quarters of the respondents with CSP had CRP (75.8%) and approximately one quarter had CWP (24.2%).

Mean EQ-5D scores were significantly lower among individuals with CSP (0.74 ± 0.2) than among individuals without CSP (0.87 ± 0.17; P < 0.001). Similarly, self-rated health scores were significantly lower among individuals with CSP (65.2 ± 21.5) than among individuals without CSP (78.8 ± 18.8; P < 0.001).

### Factors associated with chronic back pain

The following variables were selected for bivariate analysis: sex, number of comorbidities, symptoms consistent with anxiety, education, symptoms consistent with depression, age, smoking and occupational physical activity (Table 2).

**Table 2:**
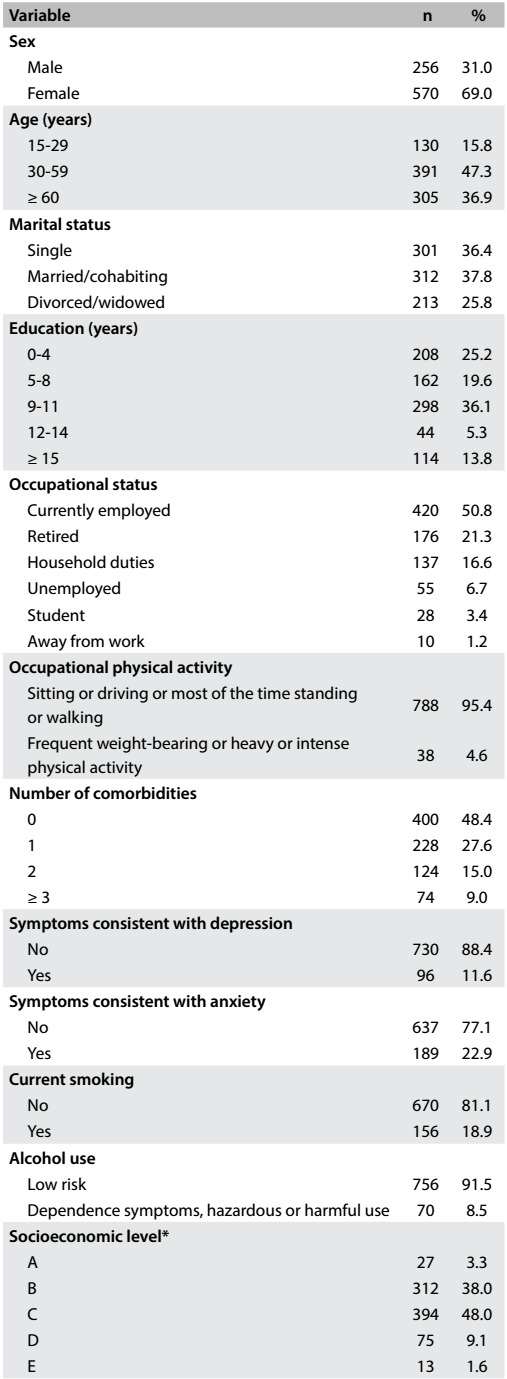
Univariate analysis on the association between chronic spinal pain and associated factors

*χ^2^ for trend; ^†^5 individuals without data. PR = prevalence ratio; CI = confidence interval.

Occupational physical activity was dichotomized in the multivariate analysis to increase its statistical power. In the final model, sex, age, education, anxiety symptoms and occupational physical activity were independently associated with CSP. The prevalence of CSP was higher among women, individuals aged 30 years or older, individuals who had low education, those who had anxiety symptoms and those who reported performing hard physical activity during the workday (Table 3).

**Table 3:**
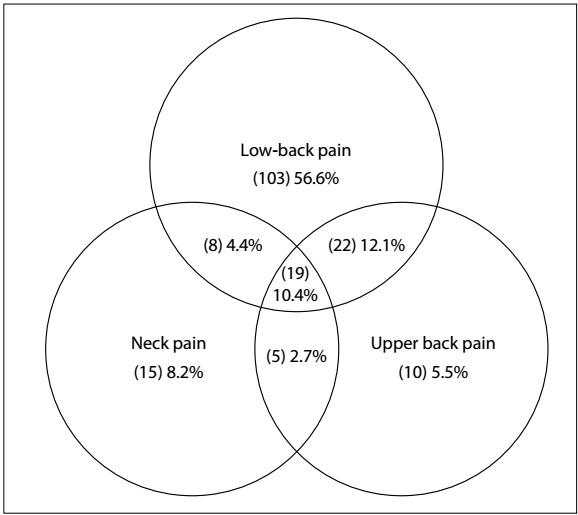
Prevalence ratios for variables independently associated with chronic spinal pain through the Cox multivariate regression model with robust variance estimation

PR_cr_ = crude prevalence ratio; PR_adj_ = adjusted prevalence ratio; CI = confidence interval. *χ^2^ for trend.

## DISCUSSION

### Prevalence and characteristics of chronic back pain

This study found that the estimated prevalence of chronic spinal pain was 22.0% (95% CI: 19.3-25.0%) in a sample of the population of São Paulo, Brazil. The separate prevalence estimates for low-back pain, upper back pain and neck pain were 18.4, 6.8 and 5.7%, respectively. Five factors were independently associated with CSP: female sex, age ≥ 30 years, education ≤ 4 years, anxiety symptoms and regular weight-bearing or heavy or intense physical activity during the workday. The prevalences of chronic regional pain (CRP) and chronic widespread pain (CWP) among individuals with CSP were 16.7% and 5.3%, respectively. In addition, self-rated quality of life and health were significantly worse among individuals with CSP than among individuals without CSP.

Most epidemiological studies have investigated the prevalence of neck pain, upper back pain, or low-back pain separately, and few studies have estimated the prevalence of chronic pain in the entire spine. Comparisons of epidemiological data on the prevalence of chronic pain in the cervical, thoracic and lumbar regions may be hindered by the lack of studies that consider the spine as a functional unit.^46^

The estimated prevalence of CSP found in this study was similar to that reported by a recent study conducted in Brazil, which found a prevalence of 19%.^17^ To our knowledge, no other Brazilian studies have examined the prevalence of chronic pain in the spine, considering the lumbar, thoracic and cervical spine as a single functional unit. The prevalence of chronic pain reported in our study for different spinal regions was similar to that reported in epidemiological surveys that categorized CSP into low-back pain, neck pain and upper back pain.^1^^,^^11^^,^^12^^,^^16^

The mean duration of pain of 6.6 ± 8.6 years was similar to the duration reported by other epidemiological surveys on the prevalence of chronic neck pain or low-back pain, which also indicated pain of long duration.^47^^,^^48^^,^^49^ The prevalence of CWP among individuals with CSP in this study (5.3%; 95% CI: 3.8-6.9%) was slightly lower than the values reported by other studies on the prevalence of chronic pain in the general population, in which the prevalence estimates for CWP have ranged from 10-13%.^50^^,^^51^ A recent study on the prevalence of widespread pain among female primary healthcare patients reported that 28% of women with chronic low-back pain had CWP,^52^ higher than the prevalence of CWP among individuals with CSP in the present study.

### Multivariate analysis

Female sex was independently associated with CSP. This finding is consistent with several epidemiological surveys on the prevalence of back pain and chronic pain.^14^^,^^17^^,^^18^^,^^19^^,^^46^^,^^48^^,^^53^^,^^54^^,^^55^ The greater prevalence of pain among women than among men may be related to cognitive and social factors. Moreover, the higher prevalence of pain among women may be a result of reporting bias, given that several studies have suggested that women are more likely to report pain than men.^56^

The prevalence of CSP was higher among individuals aged 30 years or older and was lower in the 60+ age group than in the 30 to 59-year age group. Several studies have reported that there is greater prevalence of chronic pain with increasing age.^8^^,^^10^^,^^11^^,^^12^^,^^13^^,^^14^^,^^15^ An increase in the prevalence of CSP with age has been attributed to several factors, including the increased number of comorbidities and the presence of age-related changes in the musculoskeletal system.^57^^,^^58^ Conversely, some studies have reported a slight reduction in the prevalence of low-back and neck pain after the seventh decade of life.^18^^,^^59^ The reasons for this decline of pain remain unclear, but it is possibly related to reporting bias, because back pain may be perceived as a natural part of growing older, as other age-related diseases become apparent, thus leading to underreporting of pain.^60^

Four years of education or less was independently associated with CSP. Conversely, we did not find any association between socioeconomic status and CSP. Two epidemiological surveys conducted in Brazil found that chronic pain was associated with education level, but not with socioeconomic status.^6^^,^^61^ Several studies have found an association of general chronic pain, and CSP in particular, with low education. ^8^^,^^10^^,^^11^^,^^12^^,^^13^^,^^14^^,^^15^^,^^18^^,^^19^^,^^20^^,^^23^ On the other hand, others have shown that less educated people are more likely to be affected by disabling back pain.^62^^,^^63^

Symptoms of anxiety and depression were positively associated with CSP. However, only anxiety symptoms remained independently associated with CSP in the multivariate model. The prevalence of symptoms consistent with anxiety (22.9%) or depression (11.6%) among individuals with CSP in our study was comparatively higher than estimates from other studies on chronic spinal pain. A multicenter study on mental disorders among individuals with chronic back or neck pain reported prevalences of depression and anxiety ranging from 2.5 to 15.7% and from 0.5 to 8.7%, respectively.^64^ Unlike in our study, an association between chronic pain and depression has been reported more frequently than an association between chronic pain and other emotional disorders, including anxiety.^64^

Intense physical activity or frequent weight-bearing during the workday were independently associated with CSP. Similar findings have been reported for low-back pain.^10^^,^^18^^,^^65^ Eriksen et al.^10^ reported that individuals with jobs that required intense physical effort and frequent weight-bearing activities were more likely to be affected by chronic pain than those with a sedentary occupation (OR: 2.2; 95% CI: 1.6-3.1). Conversely, other studies have suggested that mechanical factors such as lifting and carrying do not play a major role in the pathophysiology of back pain.^66^

### Study limitations

The limitations of the current study need to be noted. Firstly, the cross-sectional design cannot be used to infer a causal relationship between the factors studied and back pain. The sample size was estimated to calculate the prevalence of CSP and therefore it might not have been sufficient to identify associated factors. Secondly, because we sampled individuals from a specific region of São Paulo, it may not be possible to directly extrapolate our results to the entire population of the city. Additionally, the proportion of female respondents (69.0%) was higher than the proportion of women in the source population (58.2%). A higher proportion of women than of men has often been observed in population-based studies on chronic pain^16^^,^^54^^,^^61^^,^^67^^,^^68^ and back pain,^46^^,^^13^^,^^16^^,^^17^^,^^18^ which may lead to overestimation of the prevalence of pain. In our study, the higher proportion of women in the sample can be explained by the fact that 59% of the source population consisted of women. Moreover, most households were composed of one or two members only, and 69% of the members of these households were women. Thus, when a selected man was not interviewed, he was more likely to be replaced by a woman in a subsequent draw.

The strengths of the current study should also be noted. We used a rigorous method for participant selection. Our use of a sample from a population registered with a healthcare unit enabled us to gain access to sociodemographic information for proper planning of data collection. Telephone and letter-based interviews are the two most common types of interview used in epidemiological surveys on the prevalence of back pain, whereas home interviews are rarely used.^15^^,^^18^^,^^20^ Our use of home interviews may have improved the reliability of data collection. For each household, we were careful to make home visits at different times of the day and on different days of the week, including weekends, in an attempt to meet with participants who worked. The repeated visits resulted in a relatively high response rate (74.5%) for eligible individuals. Our use of five validated health-related quality of life instruments (depression, anxiety, alcohol use, smoking, quality of life and socioeconomic status) provided reliable data on the factors associated with CSP.

## CONCLUSION

This was the first epidemiological study to estimate the prevalence of chronic spinal pain in the largest city in Latin America. The prevalence of CSP in our sample was 22.0%. The factors independently associated with the outcome variable were female sex, age 30 years and older, low education level, anxiety symptoms and high occupational physical activity. Our suggestions for future research include a more detailed investigation of subgroups of people with chronic spinal pain, in order to identify those who are more likely to develop more severe conditions or who have greater demand for healthcare services.
